# Impact of Specialized Versus Non-Specialized Acute Hospital Care on Survival Among Patients With Acute Incomplete Traumatic Spinal Cord Injuries: A Population-Based Observational Study from British Columbia, Canada

**DOI:** 10.1089/neu.2022.0496

**Published:** 2023-11-30

**Authors:** Marcel F. Dvorak, Nathan Evaniew, Melody Chen, Zeina Waheed, Naama Rotem-Kohavi, Nader Fallah, Vanessa K. Noonan, Charles Fisher, Raphaële Charest-Morin, Nicolas Dea, Tamir Ailon, John Street, Brian K. Kwon

**Affiliations:** ^1^ Combined Neurosurgery and Orthopaedic Spine Program, University of British Columbia, Vancouver, British Columbia, Canada.; ^2^ International Collaboration on Repair Discoveries (ICORD), Vancouver, British Columbia, Canada.; ^3^ University of Calgary Spine Program, University of Calgary, Calgary, Alberta, Canada.; ^4^ McCaig Institute for Bone and Joint Health, University of Calgary, Calgary, Alberta, Canada.; ^5^ Praxis Spinal Cord Institute, Vancouver, British Columbia, Canada.

**Keywords:** human studies, neural injury, spinal cord injury, traumatic spinal cord injury

## Abstract

Given the complexity of care necessitated after an acute traumatic spinal cord injury (SCI), it seems intuitively beneficial for such care to be delivered at hospitals with specialized SCI expertise. Demonstrating these benefits is not straightforward, however. We sought to determine whether specialized acute hospital care influenced the most fundamental outcomes after SCI: mortality within the first year of injury. We compared survival among patients with incomplete tSCI admitted to a single quaternary-level trauma hospital with a specialized acute SCI program versus those admitted to trauma hospitals without specialized acute SCI care. We performed a population-based retrospective observational cohort study using administrative and clinical data linked from multiple sources in British Columbia (BC) from 2001 to 2017. Among a cohort of 1920 patients, there were 193 deaths within one year. We failed to identify a significant overall benefit for survival after adjusting for potential confounders, and the confidence intervals (CIs) were compatible with both benefit and harm (odds ratio [OR] 1.01, 95% CI 0.17 to 6.11, *p* = 0.99). Significant associations were observed with age greater than 65 (OR 4.92, 95% CI 1.66 to 14.57, *p* < 0.01), Charlson Comorbidity Index (OR 1.61, 95% CI 1.42 to 1.83, *p* < 0.01), Injury Severity Score (OR 1.08, 95% CI 1.06 to 1.11, *p* < 0.01), and traumatic brain injury (OR 2.12, 95% CI 1.32 to 3.41, p < 0.01). Among patients with acute tSCI, admission to a hospital with specialized acute SCI care was not associated with improved overall one-year survival. Subgroup analyses, however, suggested heterogeneity of effects, with little benefit for older patients with less polytrauma and substantial benefit for younger patients with greater polytrauma.

## Introduction

Acute traumatic spinal cord injuries (tSCI) are often associated with devastating life-long functional impairments. Having an SCI affects virtually every physiological system in the body, leaving affected individuals with a significantly increased risk for death over their lifespan.^1^ Aside from the obvious loss of motor and sensory function, massive perturbations to respiratory, cardiovascular, genitourinary, and immunological systems after acute SCI create considerable complexity in the medical, surgical, and rehabilitative treatment of these trauma patients.

Such complexity intuitively justifies the approach of having patients with acute SCI treated at hospitals that have specialized expertise in managing SCI. Current evidence, however, on the potential benefits of specialized care for patients with tSCI is limited to uncontrolled studies, historical cohort studies, and studies that compared timing of transfer rather than actual treatment at specialized versus non-specialized hospitals.^2–5^ Indirect evidence from studies of regionalized trauma care, centralized subspecialty surgical care, and high-volume critical care suggest important benefits, but potential trade-offs include transport and travel, logistical complexity, infrastructure needs and capacity, and costs.^6–10^

While there are many potential benefits to being cared for in a specialized SCI center, we sought to address the most basic of benefits: survival over the first year post first tSCI hospital admission after injury. Here, we compared all-cause death among patients with acute tSCI admitted to a single specialized quaternary-level trauma hospital versus death in those admitted to trauma hospitals without specialized SCI care.

## Methods

After appropriate ethics approval, we performed a population-based retrospective observational cohort study using administrative and clinical data that were linked from multiple datasets. We have reported previously on our data sources, methods for cohort creation, variables, validation strategies, and analysis plans.^11^ Additional details are provided in Supplementary Appendix SA1.

### Patient sample

All adult patients who were treated for acute cervical, thoracic, lumbar, sacral or cauda equina tSCIs in the province of British Columbia (BC) in Canada from 2001 to 2017 were identified by scanning the Discharge Abstract Database (Hospital Separations) using International Classification of Diseases (ICD)-10 codes by Population Data BC.^12^ The tSCI case definition was validated by mapping ICD-10 CA codes to International Standards for Neurological Classification of Spinal Cord Injury (ISNCSCI) descriptions of tSCI by level and severity.^11^ The eligible patient sample represents all tSCIs over this 17-year period in a geographic population estimated to be more than 5 million.^13^

Population Data BC provides research access to linkable, longitudinal, de-identified patient-level datasets. Over the 17-year period of the study, the attitudes of the treating spine surgeons at the specialized center with regard to treatment of patients with acute tSCI did not change substantially. For example, early surgery was always advocated while use of methylprednisolone was not, and intensive multi-disciplinary rehabilitation was always provided.

We excluded patients who had an Injury Severity Score (ISS) of less than 9 (considered minor injuries).^6^ We excluded patients with sensorimotor complete (American Spinal Injury Association Impairment Scale [AIS] A) injuries because almost all individuals with such complete injuries were treated in specialized care (93% of 563), thus precluding a comparison against non-specialized care.

### Data sources

Data were linked from the Canadian Institute for Health Information Discharge Abstract Database (Hospital Separations),^12^ BC Provincial Vital Statistics Agency (Deaths),^14^ the BC Trauma Registry, Medical Services Plan (MSP) Payment Information File,^15^ MSP Consolidation File (MSP Registration and Premium Billing),^16^ and the Rick Hansen Spinal Cord Injury Registry (RHSCIR).^17,18^

### Interventions–specialized versus non-specialized acute SCI hospital care

We defined specialized acute hospital according to recommendations reported by Parent and associates^3^ (Table 1). Only one acute care hospital in BC met these criteria over the study duration, which was also the only acute care hospital in BC accredited under the Accreditation Canada SCI Standards of Care program.^2^ All other hospitals in our dataset (*n* = 11) were considered non-specialized care. Patients whose acute care occurred at a non-specialized center but whose subsequent rehabilitation occurred at a specialized SCI rehabilitation center were analyzed in the non-specialized acute hospital care group.

**Table 1.** Components of Specialized Acute Hospital Care for Patients With Acute Traumatic Spinal Cord Injuries According to Recommendations Reported by Parent and Coworkers^3^

1. Location within or in close proximity to a Level 1 trauma center
2. 24-h on call availability of a dedicated spine surgery team
3. 24-h rapid access to magnetic resonance imaging and an operating room
4. A dedicated “spinal unit” for patient care that involves a physical space with a wide range of multi-disciplinary specialists (spine surgeons, physiatrists, physiotherapists, occupational therapists, nurse coordinators, social workers, psychologists)
5. A streamlined referral partnership to an in-patient spinal cord injury rehabilitation center

### Statistical analysis

We present unadjusted survival using Kaplan-Meier curves. We tested for unadjusted and adjusted associations between type of care and the primary outcome event of death at one year from the time of first tSCI admission after injury using univariate and multiple logistic regression. Candidate variables were initially selected based on clinical importance, and adjusted models. Candidate variables were: age, sex, Charlson Comorbidity Index, ISS, spine surgery, traumatic brain injury, injury level, transfer from another hospital, and time to admission.^1,3,4,6,18–22^

We evaluated model fit using the coefficient of determination (Nagelkerke R^2^), and we also performed Hosmer-Lemeshow “goodness-of-fit” tests, which showed satisfactory performance for all models. Odds Ratios (ORs) are reported with 95% Confidence Intervals (CIs). We removed the spine surgery variable from our final models because it was highly colinear with specialized care.

We performed *a priori* subgroup analyses investigating the effects of specialized care among patients with age <65 years and ISS of ≥16 (major trauma) and ≥25 (critically injured^19^) because prior literature suggested differential prognosis among these patients.^6,20–22^ We performed secondary analyses using Cox regression to account for censoring and propensity-score matching to alternatively control for potential confounders. Propensity score matching used a 1:1 greedy technique with the same variables as the logistic regression models (age, sex, Charlson Comorbidity Index, ISS, surgery, traumatic brain injury [TBI], injury level, transfer from another hospital, and time to admission).

Caliper distance was 0.25 and no particular variables were set to be exact matches between groups. Mortality rates between matched groups were compared using a chi-square test. We also report on death at 30 days as a secondary outcome.

All tests of significance were two-tailed and *p* values <0.05 were considered statistically significant. Missing data were managed by substitution from other data sources within the linked cohort when available. We report Relative and Absolute Risk Reductions (RRR and ARR) and Numbers Needed to Treat (NNT = 1/ARR) to prevent one death to aid interpretation of treatment effects, based on the propensity score matched analyses. Standard Differences are reported for continuous variables. Cells with less than five events were not reported to protect privacy. We used SAS software (version 9.4, SAS Institute Inc.) and Microsoft Excel (version 16.51, Microsoft Corp.).

## Results

**FIG. 1.**
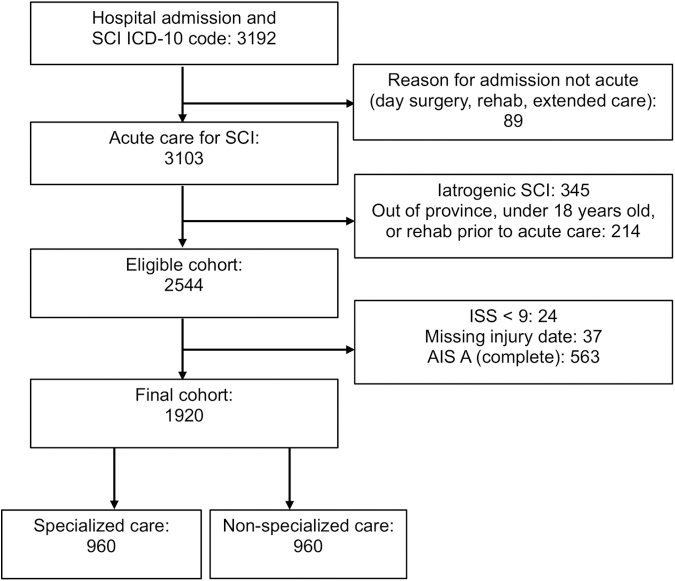
Identification of the study cohort: 1920 patients with acute traumatic spinal cord injuries (SCI) admitted to specialized or non-specialized care in the province of British Columbia from 2001 to 2017. ICD, International Classification of Diseases; ISS, Injury Severity Score; AIS, American Spinal Injury Association Impairment Scale.

We identified an eligible cohort of 2544 patients who sustained acute tSCI in BC from 2001 to 2017. Of these, we excluded 563 who had AIS A injuries, 24 who had an ISS <9, and 37 whose date of injury or admission could not be identified, yielding a final study sample of 1920 patients (Fig. 1). Of these, 960 received specialized care and 960 received non-specialized care.

We report patients' baseline characteristics according to specialized versus non-specialized acute care in Table 2. Overall mean age was 53.8 (standard deviation [SD] 15.3), 75% were male, mean ISS was 18.7 (SD 10.0), and 17% had concurrent TBIs. The most common neurological level of injury was cervical (72%). Injury Level was missing for 60 patients (3% of the study cohort). There were significant differences between groups for each of age, sex, ISS, treatment with surgery, TBI, neurological level of injury level, and the occurrence of transfer from another hospital before admission for definitive acute care.

**Table 2.** Baseline Characteristics of 1920 Patients With Acute Traumatic Spinal Cord Injuries Admitted to Specialized (*n* = 960) or Non-Specialized Care (*n* = 960) in the Province of British Columbia from 2001 to 2017

Variable	Specialized care* n* = 960	Non-specialized care,* n* = 960	*p*
Age: mean (SD)	50.7 (18.8)	57.0 (19.2)	**<0.01**
Sex: male (%)	748 (78%)	701 (73%)	**0.01**
Charlson Comorbidity Index: mean (SD)	0.4 (1.0)	0.5 (1.0)	0.11
Injury Severity Score: mean (SD)	20.6 (9.4)	16.8 (10.3)	**<0.01**
9–16 (%)	147 (16%)	402 (42%)	
16–24 (%)	558 (58%)	408 (43%)	
25 or greater (%)	253 (26%)	146 (15%)	
Spine Surgery: yes (%)	625 (65%)	364 (38%)	**<0.01**
Traumatic Brain Injury: (%)	177 (18%)	140 (15%)	**0.02**
Injury level: (%)			**0.04**
Cervical	651 (68%)	740 (77%)	
Thoracic	125 (13%)	97 (10%)	
Lumbar/Sacral/Cauda Equina	133 (14%)	114 (12%)	
Missing/Unknown	51 (5%)	9 (1%)	
Transferred from another hospital (%)	307 (32%)	59 (6%)	**<0.01**
Time from injury to admission			0.35
24 h or less	650 (68%)	679 (71%)	
25–72 h	170 (18%)	152 (16%)	
Greater than 72 h	140 (14%)	129 (13%)	

SD, standard deviation.

Injury to admission, however, did not differ significantly. Acute care length of stay was significantly longer among patients treated at specialized care (mean 39.3 days (SD 50.9) vs. 33.0 (SD 46.6), *p* < 0.01), as was total length of stay including inpatient rehabilitation (80.1 (SD 82.0) vs. 37.2 (55.9), *p* < 0.01). Patients treated at specialized care underwent spine surgery significantly more often (65% vs. 38%; *p* < 0.01) than patients at non-specialized care. The rate of eventual discharge to home after the inpatient phases of acute and rehabilitation care was 71% among patients admitted to both specialized and non-specialized care.

**FIG. 2.**
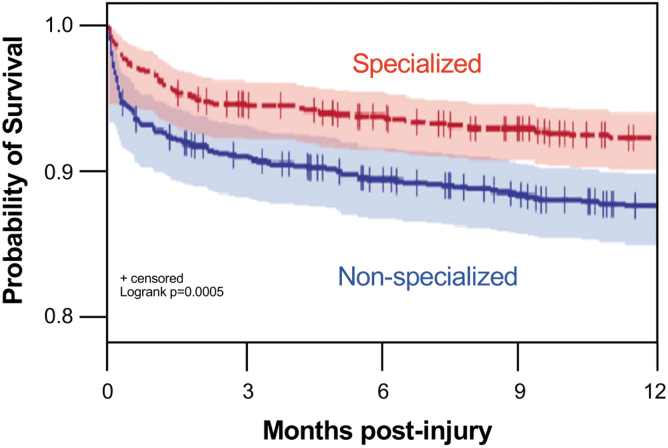
Kaplan-Meier plot or survival among 1920 patients with acute traumatic spinal cord injuries (tSCI) admitted to specialized (*n* = 960) or non-specialized care (*n* = 960) in the province of British Columbia from 2001 to 2017 with number of subjects at risk and 95% Hall-Wellner Bands.

There were 193 deaths within one year of injury across the entire cohort, representing a mortality rate of 10%. In unadjusted analyses, death was significantly lower among patients receiving specialized rather than non-specialized acute hospital care at both one year (74 [8%] vs. 119 [12%]; OR 0.59, 95% CI 0.43 to 0.80; *p* < 0.01) and 30 days (35 [4%] vs. 69 [7%]; OR 0.49, 95% CI 0.32 to 0.74; *p* < 0.01; Fig. 2 and Supplementary Appendix SA2). Causes of injury in patients who subsequently died are reported in Appendix 3, however, specific causes of death were unavailable. In comparison with those who survived, patients who died had significantly older age, more comorbidities, higher ISS, more frequent concurrent TBI, and more frequent cervical injury level (Supplementary Appendix SA3).

The results of our primary multiple logistic regression analysis are presented in Table 3. We observed significant adjusted associations between death within one year from first tSCI admission after injury and each of age greater than 65 (OR 4.92, 95% CI 1.66 to 14.57, *p* < 0.01 for age 65–74 in comparison with the reference value of 45–54; increasing ORs with increasing age thereafter), Charlson Comorbidity Index (OR 1.61, 95% CI 1.42 to 1.83, *p* < 0.01), ISS (OR 1.08, 95% CI 1.06 to 1.11, *p* < 0.01), and TBI (OR 2.21, 95% CI 1.32 to 3.41, *p* < 0.01).

**Table 3.** Adjusted Associations Between Specialized Versus Non-Specialized Care and One-Year Death Among 1920 Patients With Acute Traumatic Spinal Cord Injuries

Variable	Multiple logistic regression (*n* = 1920) Model fit: adjusted R^2^ = 0.40	
	Odds ratio	95% confidence interval
Age 18–24	0.76	0.12 – 4.72
Age 25–34	1.05	0.22 – 5.14
Age 35–44	1.22	0.29 – 5.10
Age 45–54 (reference)	-	-
Age 55–64	2.30	0.71 – 7.44
Age 65–74	4.92	1.66 – 14.57
Age 75–84	18.34	6.54 – 51.48
Age 85 or greater	26.77	8.71 – 82.33
Sex (Male)	1.41	0.92 – 2.17
Charlson Comorbidity Index (per point)	1.61	1.42 – 1.83
Injury Severity Score (per point)	1.08	1.06 – 1.11
Traumatic Brain Injury	2.12	1.32 – 3.41
Injury level – Cervical (reference)	-	-
Injury level – Thoracic	0.50	0.22 – 1.14
Injury level – Lumbar	0.78	0.32 – 1.90
Injury level – Sacral/Cauda Equina	1.88	0.37 – 9.61
Transferred from another hospital	0.84	0.48 – 1.45
Time from injury to admission <24 h (reference)	-	-
Time from injury to admission 25–72 h	1.21	0.72 – 2.05
Time from injury to admission >72 h	1.04	0.60 – 1.82
**Specialized care (vs. non-specialized)**	1.01	0.17 – 6.11

The variable “spine surgery” was excluded because it was highly correlated and colinear with type of care center. Odds Ratios less than 1 indicate decreased odds of death in association with a given variable.

When adjusting for these potential confounders within the entire cohort of 1920 patients, there was no significant difference in death between those treated in specialized care versus those in non-specialized care, and the CIs were compatible with both benefit and harm (OR 1.01, 95% CI 0.17 to 6.11, *p* = 0.99). Specialized care was also not significantly associated with reducing one-year death when the main adjusted analysis was restricted to only those patients who underwent surgery at either specialized or non-specialized care (total *n* = 989; OR 5.30, 95% CI 0.13 – 209.98, *p* = 0.37).

The results for death at 30 days were concordant (OR 0.46, 95% CI 0.05 to 3.91, *p* = 0.48). Time from injury to admission, transfer from another hospital before admission, and neurological level of injury were not significantly associated with death at one year.

We also repeated the main adjusted analysis for the whole cohort (*n* = 1920) while including an interaction term for age at injury and Charlson Comorbidity Index. We found that the interaction term was not significantly associated with one-year death (*p* = 0.44, OR 0.99, 95% CI 0.99 to 1.00, *p* = 0.44), and that the observed effect of specialized care did not change (OR 1.00, 95% CI 0.17 to 6.03, *p* = 1.00). Although the Charlson Comorbidity Index is known to be imperfect, it was the best available metric for pre-existing comorbidities in this dataset.

We present summary adjusted estimates from multiple logistic regression subgroup analyses in Table 4. After controlling for potential confounders, we observed significantly lower death at one year among patients with age less than 65 and ISS 16 or greater who were treated at specialized versus non-specialized centers (OR 0.36, 95% CI 0.15 to 0.86, *p* = 0.02). Specialized care was not significantly associated with reducing one-year death when this subgroup analysis (patients with age less than 65 and ISS 16 or greater) was restricted to only those patients who underwent spine surgery at either specialized or non-specialized care (total *n* = 572, OR 1.25, 95% CI 0.23 to 6.78; *p* = 0.80).

**Table 4.** Summary Adjusted Associations Among Subgroups of Patients With Acute Traumatic Spinal Cord Injuries Admitted to Specialized or Non-Specialized Care According to Age and Injury Severity Score

Subgroup analysis (*n*)	# of deaths	Effect of specialized versus non-specialized care on 1 year death			
		Adjusted OR	95% CI	*p*	Adjusted R^2^
Age 65 or greater (*n* = 614)	146	0.82	0.26 – 2.60	0.74	0.26
Age 65 or greater and ISS 9–16 (*n* = 169)	40	2.44	0.66 – 8.82	0.183	0.40
Age 65 or greater ISS 16 or greater (*n* = 444)	106	0.64	0.38 – 1.06	0.083	0.19
Age 65 or greater ISS 25 or greater (*n* = 102)	42	0.37	0.14 – 0.99	0.43	0.34
Age <65 (*n* = 1306)	47	1.53	0.38 – 6.07	0.55	0.36
Age <65 and ISS 9–16 (*n* = 380)	11	3.08	0.85 – 11.17	0.09	0.51
**Age <65 and ISS 16 or greater (*n* = 921)**	36	**0.36**	**0.15 – 0.86**	**0.02**	**0.45**
**Age <65 and ISS 25 or greater (*n* = 297)**	31	**0.24**	**0.09 – 0.64**	**<0.01**	**0.44**

Each summary Odds Ratio (OR) is adjusted for sex, Charlson Comorbidity Index, traumatic brain injury, neurological level of injury, transfer from another hospital, and time to admission. ORs of less than 1 indicate decreased odds of death in association with specialized care. CI, confidence interval; ISS, Injury Severity Score.

The results for death at 30 days (OR 0.21, 95% CI 0.07 to 0.62, *p* < 0.01) and among patients with age less than 65 and ISS 25 or greater at one year (OR 0.24, 95% CI 0.09 to 0.64, *p* < 0.01) and 30 days were again concordant (OR 0.19, 95% CI 0.06 to 0.60, *p* < 0.01). We report results from additional analyses that included an interaction term for age and ISS, that modeled ISS as a continuous variable, and that used Cox regression in Supplementary Appendix SA4.

**FIG. 3.**
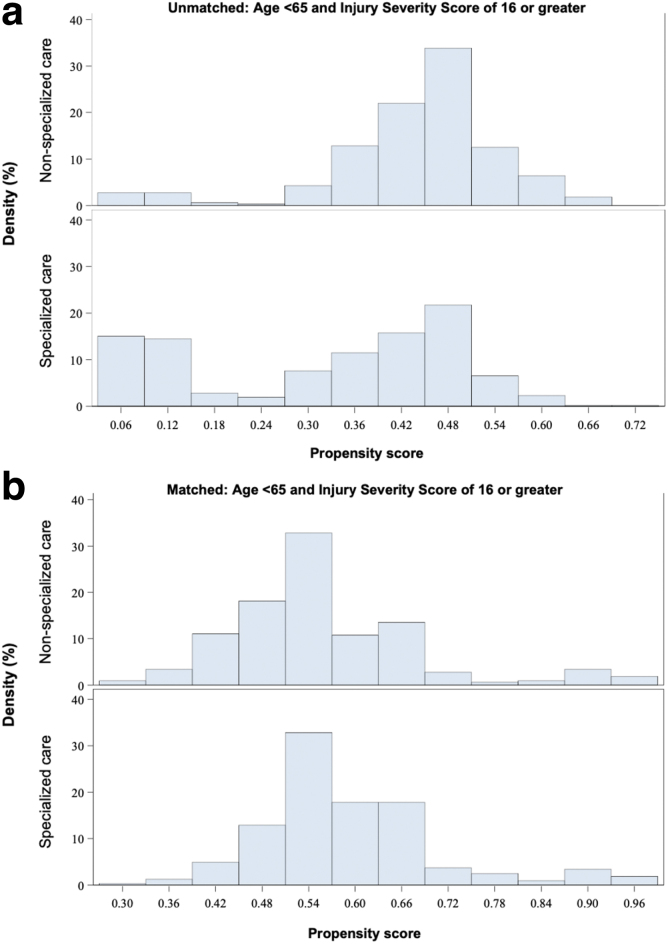
Propensity histograms show the distributions of propensity scores relative to the outcome of death among (**a**) unmatched (*n* = 921) and (**b**) matched (*n* = 652) cohorts from the subgroup of patients with acute traumatic spinal cord injuries admitted to specialized or non-specialized care with age less than 65 and Injury Severity Scores of 16 or greater.

We further tested for subgroup effects using 1:1 propensity score matching as an additional secondary analysis. We present pre- and post-matching propensity histograms in Figure 3 and baseline characteristics among matched groups of patients with age less than 65 and ISS of 16 or greater who were treated at specialized (*n* = 326) and non-specialized centers (*n* = 326) in Table 5. The only significant difference between groups was that patients treated at the specialized center again underwent spine surgery significantly more often (66% vs. 47%, *p* < 0.01).

**Table 5.** Baseline Characteristics Among the Propensity-Matched Subgroup of Patients With Acute Traumatic Spinal Cord Injuries Admitted to Specialized (*n* = 326) or Non-Specialized Care (*n* = 326) With Age Less than 65 and Injury Severity Scores of 16 or Greater

Variable	Specialized care* n* = 326	Non-specialized care,* n* = 326	*p*	Standard difference
Age: mean (SD)	44.2 (13.8)	45.4 (12.8)	0.39	0.09
Sex: male (%)	246 (76%)	263 (81%)	0.10	-
Charlson Comorbidity Index: mean (SD)	0.2 (0.7)	0.2 (0.7)	0.40	0.03
Injury Severity Score: mean (SD)	22.6 (10.3)	22.3 (10.1)	0.20	0.03
9–16 (%)	0 (0%)	0 (0%)		
16–24 (%)	242 (74%)	230 (70%)		
25–34 (%)	44 (14%)	64 (20%)		
35 or greater (%)	40 (12%)	32 (10%)		
Surgery: yes (%)	215 (66%)	152 (47%)	**<0.01**	-
Traumatic Brain Injury: (%)	64 (20%)	71 (22%)	0.50	-
Injury level:			0.49	-
Cervical (%)	238 (73%)	256 (79%)		
Thoracic (%)	42 (13%)	31 (10%)		
Lumbar (%)	37 (11%)	30 (9%)		
Sacral/Cauda Equina (%)	8 (3%)	7 (2%)		
Missing/Unknown	-	-		
Transferred from another hospital (%)	20 (6%)	23 (7%)	0.64	-
Time from injury to admission			0.32	-
24 h or less	263 (81%)	254 (78%)		
25–72 h	30 (9%)	42 (13%)		
Greater than 72 h	33 (10%)	30 (9%)		

SD, standard deviation.

**FIG. 4.**
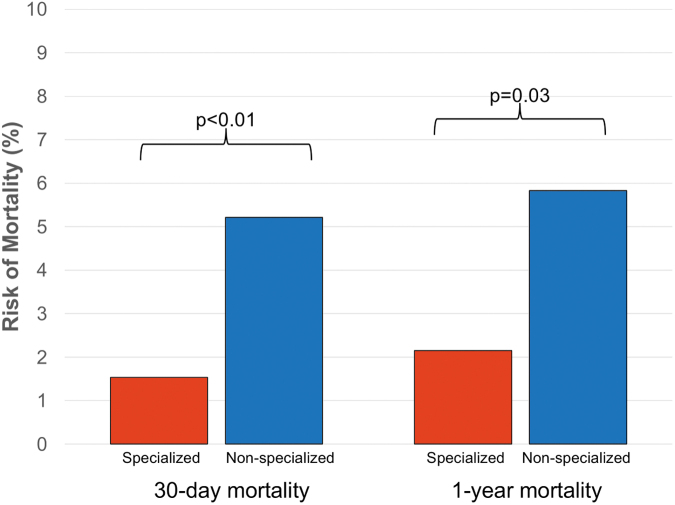
Death among the propensity-matched subgroup of patients with acute traumatic spinal cord injuries (tSCI) admitted to specialized (*n* = 326) or non-specialized care (*n* = 326) with age less than 65 and Injury Severity Scores of 16 or greater.

In this analysis, there were 26 deaths within one year, and death was significantly lower among patients treated at the specialized center rather than non-specialized acute hospital care at both one year (7 vs. 19, *p* = 0.03; RRR = 63%, ARR = 4%, NNT = 27) and 30 days (5 vs. 17, *p* < 0.01; Fig. 4). Results from a propensity-matched subgroup of patients with age less than 65 and ISS of 25 or greater were again concordant (data not shown).

## Discussion

We performed a population-based observational study to investigate the effects of specialized versus non-specialized acute hospital care on all-cause death among patients with acute incomplete tSCI. Although unadjusted analyses suggested a benefit for specialized care, analyses that controlled for potential confounding did not demonstrate a survival benefit to specialized care across the entire cohort of 1920 patients.

We interpret these findings as suggesting that individual patient factors including age in particular and concurrent polytrauma may have greater overall effects on death than type of care. Indeed, in pre-specified subgroup analyses that included only patients with younger age and greater severity of polytrauma, we consistently observed large and statistically significant relative reductions in death associated with specialized care. These results indicated that younger individuals (<65 years of age) with multiple severe injuries (ISS of 16 or greater) may have a higher chance of surviving at one year as well as at 30 days post-injury when they are treated for their acute tSCI in a specialized center.

### Relation to previous literature

The origins of specialized tSCI care are often attributed to Munro in the United States in 1936 and Guttman in England in 1944, but earlier reports describe a spinal unit founded by Wilhem Wagner at the end of the 19th century in Germany to treat miners who suffered SCIs as a result of collapsing tunnels.^23^ As described by Wiener and colleagues,^24^ care in these settings consisted mainly of turning patients regularly and draining their bladders. Since then, specialized tSCI care has evolved into dedicated units at modern tertiary or quaternary care hospitals with rapid access to imaging, critical care, neuroanesthesia, surgery, and a wide range of multi-disciplinary specialized allied healthcare providers.

Our study adds to a heterogeneous body of literature about death after SCI and, importantly, highlights the role that specialized care may play in improving survival. In a systematic review and meta-analysis of 74 studies, Chamberlain and coworkers^1^ reported a pooled one-year survival of 93% (95% CI 89% to 95%), but noted large variability. Our data yielded a similar one-year survival and confirmed associations with older age, number of comorbidities, ISS, and TBI.

While specialized trauma centers have been shown to reduce death in severely injured polytrauma patients,^6,25,26^ we are unaware of any other studies that have compared the effects of contemporary specialized and non-specialized acute hospital care on death for patients specifically with acute tSCIs. Death notwithstanding, early transfer to specialized care has been associated with decreased rates of urinary tract infections, pressure ulcers, and respiratory complications, as well as shorter acute care lengths of stay, and a greater likelihood of returning home when patients access specialized inpatient rehabilitation.^27–29^ The Consortium for Spinal Cord Medicine published a guideline statement in 2008 that included a strong recommendation in favor of specialized acute care, but this was based on mostly uncontrolled observational studies and expert opinion.^30,31^

### Implications

Our subgroup results suggest that specialized acute care may provide a survival benefit to younger patients (age <65 years) with incomplete SCIs and severe concomitant polytrauma, and that treatment of these patients at specialized centers may be preferred. This has implications on how patients with acute tSCI are triaged, especially in environments where there may be choices on where to transfer such patients from the field. These results, however, should be interpreted with caution as hypothesis-generating and in need of further confirmation because there is a possibility that they may be an artefact of multiple comparisons.

Our data suggest a referral bias because patients treated at specialized care were younger and more likely male, with higher ISS scores for polytrauma, and greater frequency of TBIs. We speculate that the spine surgery rates were lower in the non-specialized group because there was a greater proportion of patients with milder central cord syndromes in that group, but we acknowledge that our data do not directly inform about this. It is also possible that the treating physicians at non-specialized centers could have been less likely to offer spine surgery according to differences in their preferences or practice patterns.

During the study period, the specialized center maintained a “zero-refusal” policy, whereby all patients referred with tSCI were accepted for transfer regardless of age, SCI severity, pre-existing medical conditions, or other factors. As such, decisions to keep patients at non-specialized centers were made by the local physicians at those centers, who presumably were comfortable treating those patients who were not transferred.

It is possible that ageist attitudes at non-specialized centers could have contributed to not transferring older patients. It is also possible that the younger mean age in the specialized cohort simply reflects a greater demographic of patients with concomitant high-energy polytrauma (with higher ISS scores and higher rates of TBI) that required transfer for reasons other than just their SCI.

Our group has reported previously that patients with tSCI who are elderly and/or frail have substantially increased risk for death, and epidemiological studies have shown that the elderly population of patients with tSCI is a steadily growing cohort.^20–22,32^ In elderly individuals, however, our data suggest that incompletely understood factors, perhaps such as the physiological insult of tSCI superimposed on potentially limited physiological reserve, may play such a stronger role in dictating death that they overcome the potential survival benefit of being treated in a specialized center.

There may be other potential benefits to specialized care for the elderly with tSCI such as greater neurological recovery, fewer complications, or shorter length of stay, but these outcomes were not the subject of the current study. This current study confirms the increased risk of death with advanced age and supports further research in this area to determine what aspects of specialized care could be modified to potentially alter this grim prognosis after acute tSCI.

### Strengths and limitations

Many of the general strengths and limitations of population-based observational studies from administrative datasets have been discussed elsewhere.^17,33,34^ A major strength of this study is that it reports on tSCI over a 17-year period from a geographic population of approximately 5 million where there is only one specialized tSCI center, and thus has an element of generalizability that may avoid some selection biases.

Another important strength of our study is our credible implementation of subgroup analyses. Subgroup analyses are at unique potential risk for producing spurious or misleading results,^35^, but there are published criteria to judge the credibility of subgroup analyses.^36^ Our subgroup findings meet most of these criteria, although they are still limited by statistical fragility because of smaller sample sizes with fewer events.^37,38^ Relatively small event rates may have also contributed to producing wide CIs in our primary adjusted analysis.

An important limitation of our study is that we did not have the granularity in the dataset to discern among those with AIS B, C, and D severities of incomplete injury.^39^ The limitation of our analysis to those with “incomplete” AIS B, C, and D injuries is important to consider in light of the fact that even in these “less severely injured” SCIs, there appears to be a survival benefit for the younger polytrauma patients treated in specialized centers.

If non-specialized care is associated with higher deaths in these less severe SCIs, then one could hypothesize that this effect would be magnified in patients with complete paralysis, given the added medical complexity of these individuals. Given that almost all AIS A “complete” SCIs were transferred urgently to the specialized center, it was not possible to conduct an analysis of death in these complete SCIs in relation to where their acute care was delivered.

Our study is limited by lack of functional outcomes. The linked datasets did not include any measures of neurological recovery or of patient-reported function, satisfaction, and health-related quality of life. It is also possible that there have been changes to multi-system trauma triage and transportation of the injured to trauma hospitals over the 17 years of the study, but it was not possible to quantify these changes in the context of our linked datasets. This is a limitation of our study, and the potential effects of this limitation on our results and conclusions are uncertain. Additional outcomes such as readmissions and complications were beyond the scope of this article but may be the subject of future studies with this dataset.

Our study is also limited by potential residual confounding because of polytrauma. We controlled for ISS and TBI using both regression and propensity scoring, but our linked datasets did not include details about the specific injuries contributing to ISS (such as penetrating abdominal injury) or about the severity of the TBI. Our study also could not control for heterogeneity of capability among the various non-specialized centers.

We were also unable to adjust for possible differences in the timing of surgery relative to injury because of a lack of available data for this purpose. Previous studies have shown surgery earlier after tSCI to be associated with greater neurological improvement.^40^ Discharge disposition might also impact survival, but we found there was no difference in rates of discharge to home between groups in this study.

## Conclusions

Among patients with acute tSCI, admission to a hospital with specialized acute SCI care was not associated with improved one-year survival. Subgroup analyses, however, suggested heterogeneity of benefit, with little benefit for older patients with less polytrauma and substantial benefit for younger patients with greater polytrauma.

## Supplementary Material

Supplemental data

Supplemental data

Supplemental data

Supplemental data

## Supplementary Material

Supplementary Appendix SA1

Supplementary Appendix SA2

Supplementary Appendix SA3

Supplementary Appendix SA4
