# Supplementary material for: Impact of Specialized Versus Non-Specialized Acute Hospital Care on Survival Among Patients With Acute Incomplete Traumatic Spinal Cord Injuries: A Population-Based Observational Study from British Columbia, Canada
Source: J Neurotrauma. 2023 Nov 30;40(23-24):2638–47. doi: 10.1089/neu.2022.0496 (PMC10698776; doi:10.1089/neu.2022.0496)
Supplement: Supplemental data [file Suppl_AppendixSA1.docx]

**Supplementary APPENDIX SA1. Supplementary methods**

### **Data sources**

*Population Data BC:* Provincial identifiable data holdings in BC are held through Population Data BC (1). Population Data BC enables linkage between data sets to provide provincial, longitudinal deidentified health data at the patient-level. For data extraction, all admissions for individuals who were admitted to a BC hospital with a tSCI diagnosis code between January 1, 1995 and December 31, 2017 were included, using scanned hospital Discharge Abstract Database metadata.

*Rick Hansen Spinal Cord Injury Registry (RHSCIR):* A prospective pan-Canadian clinical registry of people with SCI. Across the country, there are 30 acute and rehabilitation sites in 15 cities in 9 of the 10 provinces. Patients' data are collected throughout their journey, including injuries, social determinants, pre-hospital/acute/rehabilitation care, and outcomes. The Praxis Spinal Cord Institute (formerly the Rick Hansen Institute) sponsors the study and manages the national data. The RHSCIR data in BC is collected from one Hospital and one Rehabilitation Center that provide specialized care for tSCI. For this study, data included was between April 1, 2004 – December 31 2017 (2). Until the launch of RHSCIR, a clinical quality improvement database named *VerteBase* collected information on patients admitted to the specialized care Hospital. Data was collected between January 01 1994 and December 31 2004. Additional dataset used is Vancouver Spine Database (QISpine) which includes data from Vancouver General Hospital between April 01 2013 – December 31 2017.

*BC Trauma Registry (BCTR):* This dataset captures data on patients admitted to 11 designated level 1, 2, and 3 trauma hospitals in the province (3). All admissions that have an Abbreviated Injury Severity code for tSCI between Jan 1, 1995 – Dec 31, 2017 were included for participating facilities

According to Accreditation Canada’s trauma distinction standards (From: [http://www.phsa.ca/our-services/programs-services/trauma-services-bc](about:blank)) the above centres are included in the dataset:

| **Level 1 centres** |
| --- |
| BC Children’s Hospital – Vancouver (Provincial Health Services Authority) |
| Royal Columbian Hospital – New Westminster (Fraser Health) |
| Vancouver General Hospital (Vancouver Coastal Health)* |
| **Level 2 centres** |
| Kelowna General Hospital (Interior Health) |
| Royal Inland Hospital – Kamloops (Interior Health) |
| Royal Jubilee Hospital – Victoria (Island Health) |
| Victoria General Hospital – Victoria (Island Health) |
| **Level 3 centres** |
| Abbotsford Regional Hospital & Cancer Centre (Fraser Health) |
| Lions Gate Hospital – North Vancouver (Vancouver Coastal Health) |
| Nanaimo Regional General Hospital (Island Health) |
| St. Paul’s Hospital – Vancouver (Providence Health Care) |
| University Hospital of Northern BC – Prince George (Northern Health) |

* Vancouver General Hospital was the only acute care hospital in BC met criteria for specialized acute SCI care over the study duration, which was also the only acute care hospital in BC accredited under the Accreditation Canada SCI Standards of Care program

For additional information on Data sources please refer to Noonan et al 2020 (4).

### **Variables**

Variables extracted from the database for this study in relation to *Patient Characteristics* (at admission) include age at injury (18 years and above), sex, rural vs. urban (residential location at time of injury) and Charlson Comorbidity Index. Variables related to *Injury Details* include severity of injury (complete vs. incomplete), neurological level of injury (cervical, thoracic, lumbar, sacral/cauda equina), completeness and neurological level of injury, paraplegia vs. tetraplegia, mechanism of injury, Injury Severity Score (ISS) (<16, 16-24, 25-34, >=35, and ISS continuous), TBI yes/no (Traumatic Brain Injury) using ICD codes for TBI, transferred to care centers (directly admitted to a care center vs transferred to a care center, time from injury to first admission (t<=24h, 25h<t<72h, t>72h). Variables related *Acute Care Factors* include spine surgery (yes/no). *Primary Outcome* reported was One-year mortality post-injury.

### **Diagnostic codes**

ICD-10 Diagnosis codes used for tSCI

| **ICD – 10 codes for traumatic SCI (from 2001/2002 onward)** | |
| --- | --- |
| S14.0 | Concussion and oedema of cervical spinal cord |
| S14.10 | Complete lesion of cervical spinal cord |
| S14.11 | Central cord lesion of cervical spinal cord |
| S14.12 | Anterior cord syndrome of cervical spinal cord |
| S14.13 | Posterior cord syndrome of cervical spinal cord |
| S14.18 | Other injuries of cervical spinal cord |
| S14.19 | Unspecified lesion of cervical spinal cord |
| S24.0 | Concussion and oedema of thoracic spinal cord |
| S24.10 | Complete lesion of thoracic spinal cord |
| S24.11 | Central cord lesion of thoracic spinal cord |
| S24.12 | Anterior cord syndrome of thoracic spinal cord |
| S24.13 | Posterior cord syndrome of thoracic spinal cord |
| S24.18 | Other injuries of thoracic spinal cord |
| S24.19 | Unspecified lesion of thoracic spinal cord |
| S34.0 | Concussion and oedema of lumbar spinal cord |
| S34.10 | Complete lesion of lumbar spinal cord |
| S34.11 | Central cord lesion of lumbar spinal cord |
| S34.12 | Anterior cord syndrome of lumbar spinal cord |
| S34.13 | Posterior cord syndrome of lumbar spinal cord |
| S34.18 | Other injuries of lumbar spinal cord |
| S34.19 | Unspecified lesion of lumbar spinal cord |
| S34.30 | Laceration of cauda equine |
| S34.38 | Other and unspecified injury of cauda equine |
| T06.0 | Injuries of brain and cranial nerves with injuries of nerves and spinal cord at neck level |
| T06.1 | Injuries of nerves and spinal cord involving other multiple body regions |

### **Data Validation**

### Data validation was done using various steps (4). The central derived dataset created for BC linked dataset contained commonly used derived variables (e.g., index SCI visit, first ISNCSCI exam, etc.). Venn diagrams was created to check the reliability of the linked cohort sizes. Additionally, the cohort was validated against available literature whereas derivations of key variables using administrative data such as index SCI visit and indirect versus direct admission to a specialized center were validated against RHSCIR data (4).

### **Data Linkage**

For data linkage, PopData combines a deterministic and probabilistic linkage and clerical review for competing matches (5). Data was handled in accordance with Population Data BC's security and privacy policies. Date of birth and personal health number have been used to link the data. Patients' deidentified linked data were uploaded to Population Data BCs Secure Research Environment (SRE). The University of British Columbia (on behalf of Population Data BC) and Vancouver Coastal Health (on behalf of the Vancouver Spine Database that includes VerteBase and RHSCIR) signed an Information Sharing Agreement. In addition, permission was obtained from the respective data stewards for RHSCIR, VerteBase, Ministry of Health, Vital statistics, WorkSafeBC and BC Trauma Registry. An agreement between Population Data BC and the principal investigator was also required before linking the datasets.

### **Additional information on Data Flow**

PopDataBC inclusion criteria for extract included all admissions for individuals who were admitted to a BC hospital with a tSCI ICD-10 diagnosis code between January 2001 and December 31, 2017 (using data from the DAD).

Individuals with acute care index admission with a tSCI diagnosis corresponding to a diagnosis type M, 1, 3, W, X, Y were included (M (Main diagnosis), 1 (pre-admit comorbidity), 3 (secondary diagnosis), W/X/Y (service transfer diagnosis)).

Individuals who did not have any admission with a tSCI diagnosis code or individuals with a tSCI admission of diagnosis type 2 (post-admit comorbidity) were excluded (N=89).

Additional exclusions included tSCI causes of injury that were iatrogenic (N=345), individuals with only out-of-province acute care for tSCI **OR** younger than 18 years at time of injury **OR** visited rehab site for tSCI prior to acute site (N=214).

Individuals with ISS<9 (N=24) or without injury date (derived using MSP data set) (N=37) or with a complete injury (AIS-A) (N=563) were excluded as well.

This resulted in the final cohort of 1920 individuals of which 960 were in specialized care and 960 were in non-specialized care.

The table below shows distribution of individuals included in the analysis per year following exclusion criteria for specialized and non-specialized care:

| Admission Year for First Acute tSCI | Specialized | Non-Specialized |
| --- | --- | --- |
| 2001 | 37 | 24 |
| 2002 | 49 | 42 |
| 2003 | 55 | 45 |
| 2004 | 40 | 61 |
| 2005 | 55 | 57 |
| 2006 | 66 | 53 |
| 2007 | 59 | 51 |
| 2008 | 65 | 53 |
| 2009 | 57 | 70 |
| 2010 | 44 | 55 |
| 2011 | 64 | 50 |
| 2012 | 53 | 79 |
| 2013 | 58 | 63 |
| 2014 | 62 | 76 |
| 2015 | 76 | 53 |
| 2016 | 69 | 65 |
| 2017 | 51 | 63 |
| Total | 960 | 960 |

| **Instrument for assessing the Credibility of Effect Modification Analyses (ICEMAN) (6) - https://www.iceman.help/overview** |
| --- |
| 1: Was the direction of the effect modification correctly hypothesized a priori?  Yes – we hypothesized that a greater mortality benefit for specialized care would be seen among younger patients with greater severity of polytrauma. |
| 2: Was the effect modification supported by prior evidence?  Yes – we hypothesized differential prognosis with worse outcomes among elderly patients according to Ahn et al., Inglis et al., and Banaszek et al.; and that the benefit of specialized care would be greater among patients with greater severity of polytrauma according to MacKenzie et al. (6-9) |
| 3. Does a test for interaction suggest that chance is an unlikely explanation of the apparent effect modification?  Yes – Appendix 4 (Table 1) presents adjusted associations between specialized versus non-specialized care and one year mortality, with an interaction term for age and ISS that is statistically significant. |
| 4. Did the authors test only a small number of effect modifiers or consider the number in their statistical analysis?  Yes – We tested only for the effects of age and severity of polytrauma. |
| 5. If the effect modifier is a continuous variable, were arbitrary cut points avoided?  Probably yes – the analyses of age and ISS were based on pre-specified cut points (Age <65 and ISS 16 or greater) |
| 6 Optional: Are there any additional considerations that may increase or decrease credibility?  Yes, probably increase – a sensitivity analysis suggested robustness to relevant assumptions (propensity score matching); the effect modification persisted after adjustment for other potential effect modifiers (multivariate logistic regression). |
| **7. How would you rate the overall credibility of the proposed effect modification?**  **High credibility:** no response options definitely or probably reduced credibility. Very likely effect modification. |

**References**

1. Canadian Institute for Health Information [creator](2019): Discharge Abstract Database (Hospital Separations). V2. Population Data BC [publisher]. Data Extract. MOH(2019). <http://www.popdata.bc.ca/data>

2. Noonan VK, Kwon BK, Soril L, et al. The Rick Hansen Spinal Cord Injury Registry (RHSCIR): a national patient-registry. *Spinal Cord*. 2012;50(1):22–7.

3. British Columbia Ministry of Health [creator](2018): Medical Services Plan (MSP) Payment Information File. V2. Population Data BC [publisher]. Data Extract. MOH(2019). http://www.popdata.bc.ca/data

4. Noonan VK, Jaglal SB, Humphreys S, et al. Linking Spinal Cord Injury Data Sets to Describe the Patient Journey Following Injury: A Protocol. *Top. Spinal Cord Inj. Rehabil.* 2020;26(4):232–242.

5. Ark TK, Kesselring S, Hills B, McGrail KM. Population Data BC: Supporting population data science in British Columbia. Int J Popul Data Sci. 2020 Mar 26;4(2):1133. doi: 10.23889/ijpds.v5i1.1133. PMID: 32935036; PMCID: PMC7480325.

6. Schandelmaier S, Briel M, Varadhan R, Schmid CH, Devasenapathy N, Hayward RA, Gagnier J, Borenstein M, van der Heijden GJMG, Dahabreh IJ, Sun X, Sauerbrei W, Walsh M, Ioannidis JPA, Thabane L, Guyatt GH. Development of the Instrument to assess the Credibility of Effect Modification Analyses (ICEMAN) in randomized controlled trials and meta-analyses. CMAJ. 2020 Aug 10;192(32):E901-E906.

7. Ahn H, Bailey CS, Rivers CS, et al. Effect of older age on treatment decisions and outcomes among patients with traumatic spinal cord injury. *CMAJ Can. Med. Assoc. J. J. Assoc. Medicale Can.* 2015;187(12):873–880.

8. Inglis T, Banaszek D, Rivers CS, et al. In-Hospital Mortality for the Elderly with Acute Traumatic Spinal Cord Injury. *J. Neurotrauma*. 2020.

22. Banaszek D, Inglis T, Marion TE, et al. Effect of Frailty on Outcome after Traumatic Spinal Cord Injury. *J. Neurotrauma*. 2020;37(6):839–845

9. MacKenzie EJ, Rivara FP, Jurkovich GJ, et al. A national evaluation of the effect of trauma-center care on mortality. *N. Engl. J. Med.* 2006;354(4):366–378.
