# Supplementary material for: Impact of Specialized Versus Non-Specialized Acute Hospital Care on Survival Among Patients With Acute Incomplete Traumatic Spinal Cord Injuries: A Population-Based Observational Study from British Columbia, Canada
Source: J Neurotrauma. 2023 Nov 30;40(23-24):2638–47. doi: 10.1089/neu.2022.0496 (PMC10698776; doi:10.1089/neu.2022.0496)
Supplement: Supplemental data [file Suppl_AppendixSA2.docx]

**Supplementary Appendix SA2. Unadjusted outcomes**

**Figure 1.** Unadjusted mortality among 1920 patients with acute traumatic spinal cord injuries (SCI) admitted to specialized (n=960) or non-specialized care (n=960) in the province of British Columbia from 2001 to 2017. In unadjusted analyses, mortality was significantly lower among patients receiving specialized rather than non-specialized acute hospital care at both one-year (74 (8%) vs 119 (12%); OR 0.59, 95% CI 0.43 to 0.80; p<0.01) and 30-days (35 (4%) vs 69 (7%); OR 0.49, 95% CI 0.32 to 0.74; p<0.01).
