# Supplementary material for: Impact of Specialized Versus Non-Specialized Acute Hospital Care on Survival Among Patients With Acute Incomplete Traumatic Spinal Cord Injuries: A Population-Based Observational Study from British Columbia, Canada
Source: J Neurotrauma. 2023 Nov 30;40(23-24):2638–47. doi: 10.1089/neu.2022.0496 (PMC10698776; doi:10.1089/neu.2022.0496)
Supplement: Supplemental data [file Suppl_AppendixSA3.docx]

**Supplementary APPENDIX SA3. Additional information about patients who died**

**Table 1.** Causes of injury in patients who subsequently died within one year among 1920 patients with acute traumatic spinal cord injuries admitted to specialized (n=960) or non-specialized care (n=960) in the province of British Columbia from 2001 to 2017. Specific causes of death for each patient were not available.

| **Cause of injury** | **Specialized care**  **n=960** | **Non-specialized care n=960** |
| --- | --- | --- |
| Motor vehicle accidents | 11 | 18 |
| Falls/Non-transport accidents and their sequelae | 8 | 30 |
| Other causes (not specified) | 55 | 71 |
| **Total** | **74** | **119** |

**Table 2.** Baseline characteristics of 193 patients with acute traumatic spinal cord injuries who died within one year of injury versus 1727 who survived.

| **Variable** | **Died**  **n=193** | **Survived**  **n=1727** | **p-value** |
| --- | --- | --- | --- |
| Age: mean (SD) | 71.62 (17.26) | 51.88 (18.44) | <0.01 |
| Sex: male (%) | 149 (77.2) | 1300 (75.3) | 0.56 |
| Charlson Comorbidity Index: mean (SD) | 1.35 (1.85) | 0.31 (0.82) | <0.01 |
| Injury Severity Score: mean (SD)  16-24 (%)  25 or greater (%) | 24.78 (18.41)  69 (35.8)  38 (19.7) | 17.98 (8.38)  897 (51.9)  235 (13.6) | <0.01 |
| Traumatic Brain Injury: (%) | 57 (29.5) | 260 (15.1) | <0.01 |
| Injury level: (%)  Cervical  Thoracic  Lumbar/Sacral/Cauda Equina  Missing/Unknown | 160 (82.9)  14 (7.3)  13 (6.7)  6 (3.1) | 1231 (71.3)  208 (12)  234 (13.6)  54 (3.1) | 0.02 |
| Transferred from another hospital (%) | 33 (17.1) | 333 (19.3) | 0.46 |
| Time from injury to admission  24 hours or less  25-72 hours  Greater than 72 hours | 136 (70.5)  26 (13.5)  31 (16.1) | 1193 (69.1)  243 (14.1)  291 (16.9) | 0.93 |
