# Supplementary material for: Impact of Specialized Versus Non-Specialized Acute Hospital Care on Survival Among Patients With Acute Incomplete Traumatic Spinal Cord Injuries: A Population-Based Observational Study from British Columbia, Canada
Source: J Neurotrauma. 2023 Nov 30;40(23-24):2638–47. doi: 10.1089/neu.2022.0496 (PMC10698776; doi:10.1089/neu.2022.0496)
Supplement: Supplemental data [file Suppl_AppendixSA4.docx]

Supplementary **APPENDIX SA4. Additional analyses**

**Table 1.** Adjusted associations between specialized versus non-specialized care and one year mortality among 1920 patients with acute traumatic spinal cord injuries, with an interaction term for age and ISS.

| **Variable** | **Multiple logistic regression**  **Model fit: Adjusted R^2^ = 0.40** | | |
| --- | --- | --- | --- |
|  | **Odds Ratio** | **95% Confidence Interval** | **p-value** |
| Age | 1.12 | 1.09 – 1.15 | **<0.01** |
| Sex (Male) | 1.38 | 0.90 – 2.11 | 0.14 |
| Charlson Comorbidity Index (per point) | 1.59 | 1.40 – 1.81 | **<0.01** |
| Injury Severity Score (per point) | 1.21 | 1.14 – 1.28 | **<0.01** |
| Traumatic Brain Injury | 2.33 | 1.46 – 3.71 | **<0.01** |
| Injury level – Cervical (reference) | - | - | - |
| Injury level – Thoracic | 0.45 | 0.19 – 1.06 | 0.07 |
| Injury level – Lumbar | 0.74 | 0.05 – 58.98 | 0.75 |
| Injury level – Sacral/Cauda Equina | 1.69 | 0.31 – 9.12 | 0.54 |
| Transferred from another hospital | 0.84 | 0.48 – 1.45 | 0.53 |
| Time from injury to admission <24h (reference) | - | - | - |
| Time from injury to admission 25-72h | 1.27 | 0.75 – 2.14 | 0.37 |
| Time from injury to admission >72h | 0.96 | 0.55 – 1.69 | 0.90 |
| Specialized care (vs non-specialized) | 1.59 | 0.16 – 15.68 | 0.69 |
| **Age * ISS** | **0.998** | **0.997 - 0.999** | **0.0001** |

**Table 2.** Adjusted associations between specialized versus non-specialized care and one year mortality among 1920 patients with acute traumatic spinal cord injuries, with an interaction term for age and care type.

| **Variable** | **Multiple logistic regression**  **Model fit: Adjusted R^2^ = 0.19** | | |
| --- | --- | --- | --- |
|  | **Odds Ratio** | **95% Confidence Interval** | **p-value** |
| Age 18-24 | 0.76 | 0.12 - 4.72 | 0.77 |
| Age 25-34 | 1.05 | 0.22 - 5.14 | 0.95 |
| Age 35-44 | 1.22 | 0.29 - 5.10 | 0.79 |
| Age 55-64 | 2.30 | 0.71 - 7.44 | 0.16 |
| Age 65-74 | 4.92 | 1.66 - 14.57 | **<0.01** |
| Age 75-84 | 18.34 | 6.54 - 51.48 | **<0.01** |
| Age >=85 | 26.77 | 8.71 - 82.33 | **<0.01** |
| Sex (Male) | 1.41 | 0.92 – 2.17 | 0.12 |
| Charlson Comorbidity Index (per point) | 1.61 | 1.42 – 1.83 | **<0.01** |
| Injury Severity Score (per point) | 1.08 | 1.06 – 1.11 | **<0.01** |
| Traumatic Brain Injury | 2.12 | 1.31 – 3.41 | **<0.01** |
| Injury level – Cervical (reference) | - | - | - |
| Injury level – Thoracic | 0.50 | 0.22 – 1.14 | 0.10 |
| Injury level – Lumbar | 0.78 | 0.32 – 1.90 | 0.58 |
| Injury level – Sacral/Cauda Equina | 1.88 | 0.37 – 9.61 | 0.45 |
| Transferred from another hospital | 0.84 | 0.48 – 1.45 | 0.53 |
| Time from injury to admission <24h (reference) | - | - | - |
| Time from injury to admission 25-72h | 1.21 | 0.72 – 2.05 | 0.47 |
| Time from injury to admission >72h | 1.04 | 0.60 – 1.82 | 0.89 |
| Specialized care (vs non-specialized) | 1.01 | 0.17 – 6.11 | 0.99 |
| **Age 18-24 * Specialized care** | 2.66 | 0.21 - 33.05 | 0.45 |
| **Age 25-34 * Specialized care** | 0.48 | 0.03 - 8.12 | 0.61 |
| **Age 35-44 * Specialized care** | 1.45 | 0.17 - 12.52 | 0.74 |
| **Age 55-64 * Specialized care** | 1.01 | 0.16 - 6.22 | 1.00 |
| **Age 65-74 * Specialized care** | 1.31 | 0.24 - 7.19 | 0.76 |
| **Age 75-84 * Specialized care** | 1.22 | 0.23 - 6.37 | 0.81 |
| **Age >=85 * Specialized care** | 0.93 | 0.15 - 5.97 | 0.94 |

**Table 3.** Adjusted associations between specialized versus non-specialized care and one year mortality among 1920 patients with acute traumatic spinal cord injuries, with Injury Severity Score (ISS) analyzed as a continuous variable in the age groups of younger than 65 (n=1306) versus 65 or greater (n=614).

**Age younger than 65 (n=1306)**

| **Variable** | **Multiple logistic regression**  **Model fit: Adjusted R^2^ = 0.36** | | |
| --- | --- | --- | --- |
|  | **Odds Ratio** | **95% Confidence Interval** | **p-value** |
| Sex (Male) | 1.59 | 0.64 – 3.96 | 0.31 |
| Charlson Comorbidity Index (per point) | 1.77 | 1.43 – 2.20 | **<0.01** |
| Injury Severity Score (per point) | 1.11 | 1.07 – 1.15 | **<0.01** |
| Traumatic Brain Injury | 3.01 | 1.46 – 6.21 | **<0.01** |
| Injury level – Cervical (reference) | - | - | - |
| Injury level – Thoracic | 0.13 | 0.02 – 0.85 | **0.03** |
| Injury level – Lumbar | 0.33 | 0.04 – 2.54 | 0.28 |
| Injury level – Sacral/Cauda Equina | 0.76 | 0.03 – 18.42 | 0.87 |
| Transferred from another hospital | 0.91 | 0.36 – 2.27 | 0.83 |
| Time from injury to admission <24h (reference) | - | - | - |
| Time from injury to admission 25-72h | 1.24 | 0.47 – 3.25 | 0.67 |
| Time from injury to admission >72h | 2.07 | 0.81 – 5.25 | 0.13 |
| Specialized care (vs non-specialized) | 1.55 | 0.38 – 6.35 | 0.55 |

**Age 65 or greater (n=614)**

| **Variable** | **Multiple logistic regression**  **Model fit: Adjusted R^2^ = 0.26** | | |
| --- | --- | --- | --- |
|  | **Odds Ratio** | **95% Confidence Interval** | **p-value** |
| Sex (Male) | 1.43 | 0.88 – 2.31 | 0.15 |
| Charlson Comorbidity Index (per point) | 1.51 | 1.29 – 1.77 | **<0.01** |
| Injury Severity Score (per point) | 1.06 | 1.03 – 1.09 | **<0.01** |
| Traumatic Brain Injury | 1.73 | 0.94 – 3.20 | 0.08 |
| Injury level – Cervical (reference) | - | - | - |
| Injury level – Thoracic | 0.59 | 0.27 – 1.28 | 0.18 |
| Injury level – Lumbar | 0.87 | 0.34 – 2.21 | 0.76 |
| Injury level – Sacral/Cauda Equina (no patients) | - | - | - |
| Transferred from another hospital | 0.92 | 0.48 – 1.75 | 0.79 |
| Time from injury to admission <24h (reference) | - | - | - |
| Time from injury to admission 25-72h | 1.24 | 0.68 – 2.26 | 0.49 |
| Time from injury to admission >72h | 0.79 | 0.41 – 1.53 | 0.48 |
| Specialized care (vs non-specialized) | 0.97 | 0.30 – 3.11 | 0.96 |

**Table 4.** Cox regression. Adjusted associations between specialized versus non-specialized care and one year mortality among 1920 patients with acute traumatic spinal cord injuries. Hazard Ratios less than 1 indicate decreased odds of mortality in association with a given variable.

| **Variable** | **Cox regression** | | |
| --- | --- | --- | --- |
|  | **Hazard Ratio** | **95% Confidence Interval** | **p-value** |
| Age 18-24 | 1.16 | 0.23 – 5.83 | 0.89 |
| Age 25-34 | 1.75 | 0.49 – 6.28 | 0.39 |
| Age 35-44 | 1.30 | 0.36 – 4.66 | 0.68 |
| Age 45-54 (reference) | - | - | - |
| Age 55-64 | 2.44 | 0.86 – 6.94 | 0.10 |
| Age 65-74 | 4.40 | 1.75 – 11.06 | **<0.01** |
| Age 75-84 | 11.57 | 4.91 – 27.24 | **<0.01** |
| Age 85 or greater | 19.12 | 7.66 – 47.73 | **<0.01** |
| Sex (Male) | 1.32 | 0.93 – 1.88 | 0.12 |
| Charlson Comorbidity Index | 1.32 | 1.22 – 1.43 | **<0.01** |
| Injury Severity Score | 1.06 | 1.05 – 1.07 | **<0.01** |
| Traumatic Brain Injury | 1.97 | 1.39-2.77 | **<0.01** |
| Injury level – Cervical (reference) | - | - | - |
| Injury level – Thoracic | 0.68 | 0.35 – 1.31 | 0.25 |
| Injury level – Lumbar | 0.72 | 0.36 – 1.45 | 0.35 |
| Injury level – Sacral/Cauda Equina | 1.74 | 0.42 – 7.20 | 0.45 |
| Transferred from another hospital | 0.90 | 0.57 – 1.42 | 0.65 |
| Time from injury to admission <24h (reference) | - | - | - |
| Time from injury to admission 25-72h | 1.19 | 0.77 – 1.83 | 0.43 |
| Time from injury to admission >72h | 1.07 | 0.66 – 1.72 | 0.79 |
| **Specialized care (vs non-specialized)** | 1.11 | 0.24 – 5.09 | 0.89 |

| **Subgroup analysis (n)** | **Effect of specialized versus non-specialized**  **care on 1 year mortality** | | |
| --- | --- | --- | --- |
|  | Hazard Ratio | 95% Confidence Interval | p-value |
| Age <65 and ISS 16 or greater (n=921) | 0.37 | 0.17 – 0.82 | **0.01** |
| Age <65 and ISS 25 or greater (n=297) | 0.22 | 0.09 – 0.56 | **<0.01** |
